# Partial Gene Deletions of *PMP22* Causing Hereditary Neuropathy with Liability to Pressure Palsies

**DOI:** 10.1155/2014/946010

**Published:** 2014-11-20

**Authors:** Sun-Mi Cho, Bo Young Hong, Yoonjung Kim, Sang Guk Lee, Jin-Young Yang, Juwon Kim, Kyung-A Lee

**Affiliations:** ^1^Department of Laboratory Medicine, Severance Hospital, Yonsei University College of Medicine, 50-1 Yonsei-ro, Seodaemun-gu, Seoul 120-752, Republic of Korea; ^2^Department of Rehabilitation Medicine, The Catholic University of Korea, Street Vincent's Hospital, 93-6 Ji-dong, Paldal-gu, Suwon, Gyeonggi-do 442-723, Republic of Korea; ^3^Samkwang Medical Laboratories, 9-60 Yangjae-dong, Seocho-gu, Seoul 137-887, Republic of Korea; ^4^Department of Laboratory Medicine, College of Medicine, The Catholic University of Korea, 62 Yeouido-dong, Yeongdeungpo-gu, Seoul 150-713, Republic of Korea; ^5^Department of Laboratory Medicine, Yonsei University Wonju College of Medicine, 20 Ilsan-ro, Ilsandong, Wonju, Gangwon-do 220-701, Republic of Korea; ^6^Department of Laboratory Medicine, Gangnam Severance Hospital, Yonsei University College of Medicine, 712 Eonjuro, Gangnam-gu, Seoul 135-720, Republic of Korea; ^7^Department of Rehabilitation, Institute of Neuromuscular Disease, Gangnam Severance Hospital, Yonsei University College of Medicine, 712 Eonjuro, Gangnam-gu, Seoul 135-720, Republic of Korea

## Abstract

Hereditary neuropathy with liability to pressure palsies (HNPP) is an autosomal neuropathy that is commonly caused by a reciprocal 1.5 Mb deletion on chromosome 17p11.2, at the site of the peripheral myelin protein 22 (*PMP22*) gene. Other patients with similar phenotypes have been shown to harbor point mutations or small deletions, although there is some clinical variation across these patients. In this report, we describe a case of HNPP with copy number changes in exon or promoter regions of *PMP22*. Multiplex ligation-dependent probe analysis revealed an exon 1b deletion in the patient, who had been diagnosed with HNPP in the first decade of life using molecular analysis.

## 1. Introduction

Hereditary neuropathy with liability to pressure palsies (HNPP, OMIM number 162500) is an autosomal dominant peripheral neuropathy characterized by recurrent peripheral nerve palsies or sensory loss, often following minor trauma or compression in various locations, including the brachial plexus or common peroneal, ulnar, radial, or median nerves [1]. Because the symptoms of HNPP most commonly develop in adolescence or adulthood, only a few cases of childhood-onset HNPP have been reported [2, 3]. In children, other conditions may also interfere with the diagnostic accuracy of electrophysiological testing, and molecular genetic studies are needed to confirm the disease. HNPP is caused by a deletion of the* peripheral myelin protein 22-kDa *(*PMP22*, OMIM number 601097) gene on chromosome 17p11.2, which encodes an intrinsic, tetraspan membrane glycoprotein that is expressed mainly in Schwann cells and represents an important, although minor, component of the compact myelin of the peripheral nerves [4]. Charcot-Marie-Tooth neuropathy type 1A (CMT1A) is also most often associated with a tandem 1.5 Mb duplication of* PMP22 *[5].* PMP22* is a 40 kb gene that consists of six exons, of which two alternatively transcribed exons (1a and 1b) comprise the first exon of the gene [6, 7]. In this study, we report on a HNPP patient with rare copy number changes detected by multiplex ligation-dependent probe analysis (MLPA) using improved set of probes for the* PMP22* gene.

## 2. Case Presentation

The patient was a 2-year-old boy admitted to the hospital for the evaluation of left side weakness. He was born to a 31-year-old Korean mother and a 28-year-old Indonesian father at 36-week gestational age by cesarean section for breech presentation. The brain CT showed small nodular hemorrhages in the right cerebellar hemisphere; the brain MRI revealed no evidence of abnormal findings and the brain sonogram showed only mild flaring. EEG showed no epileptiform discharge or evidence of abnormal slowing. No retinopathy of prematurity was found. A conventional chromosome study showed a normal karyotype. The nerve conduction study revealed delays in conduction velocity as measured by the compound motor action potential (CMAP) in the right peroneal nerve. A screening test for 46 metabolic disorders was negative. The* dystrophia myotonica-protein kinase *gene PCR analysis for myotonic dystrophy I and the MLPA for Duchenne muscular dystrophy and spinal muscular atrophy were also negative.

Informed consent was obtained from the guardian of the patient prior to the molecular analysis of the* PMP22*. There were an additional 100 controls selected from individuals in whom demyelinating disease was not clinically evident and informed consent was also obtained before their enrollment in the study. Genomic DNA was extracted from EDTA whole blood samples with an Easy-DNA Kit (Invitrogen, Carlsbad, CA, USA). MLPA was performed using the MLPA kit (SALSA MLPA KIT P033 CMT1, MRC Holland, Amsterdam, The Netherlands) according to the manufacturer's instructions, as described in the protocol available online (http://www.mlpa.com/WebForms/WebFormMain.aspx?Tag=_fNPBLedDVp38p-CxU2h0mQ..). MLPA fragment analysis data were generated on the ABI 3500xl system (Applied Biosystems) and analyzed using the GeneMarker software (SoftGenetics, State College, PA, USA). To determine whether the MLPA results were caused by the specific mutations in the probe binding sites, PCR and direct sequencing were performed using primers designed for all exons and flanking introns of* PMP22*. To detect any sequence variation, the sequences were compared to the reference sequences using Sequencher software (Gene Codes, Ann Arbor, MI, USA).

The MLPA analysis revealed a deletion of exons in the patient. This finding was only present in the affected patient and was not found in an analysis of 200 alleles from 100 subjects without evidence of neuromuscular disease. The possibility of the presence of mutations in the probe binding sites or in the region of deletion identified by MLPA was ruled out through direct sequencing; no mutation was identified.

## 3. Discussion

Some phenotypic variability has also been observed among the CMT1A and HNPP patients, although the relationship between genetic and phenotypic variation is unclear. The patient, in whom HNPP was identified during the first decade of life, was revealed to have a novel deletion in the promoter region (exon 1b) of* PMP22*. Since there have been only a small number of HNPP cases reported during childhood, clinical suspicion of the disease is often low and the symptoms may be overlooked and detailed testing to definitively rule out HNPP is often not performed [2, 3]. To further complicate the diagnosis, the EMG in children is rather difficult to perform, making genetic testing warranted only for patients with possible HNPP. In our patient, the first and second EMG results showed normal conduction, and only the third nerve conduction test revealed a conduction delay in the peroneal nerve suspicious of demyelinating peripheral neuropathy. This final EMG result prompted us to further carry out molecular studies to confirm the diagnosis. As mentioned in Chromik et al. [8], genetic testing for patients with clinical suspicion of HNPP even when nerve conduction study results do not prefer HNPP could help physicians to diagnose HNPP in childhood. Future work will need to examine whether the* PMP22* gene dosage is regulated by the copy number change in the promoter region as well.
