# Erratum to “Partial Gene Deletions of *PMP22* Causing Hereditary Neuropathy with Liability to Pressure Palsies”

**DOI:** 10.1155/2015/239874

**Published:** 2015-03-02

**Authors:** Sun-Mi Cho, Bo Young Hong, Yoonjung Kim, Sang Guk Lee, Jin-Young Yang, Juwon Kim, Kyung-A Lee

**Affiliations:** ^1^Department of Laboratory Medicine, Severance Hospital, Yonsei University College of Medicine, 50-1 Yonsei-ro, Seodaemun-gu, Seoul 120-752, Republic of Korea; ^2^Department of Rehabilitation Medicine, The Catholic University of Korea, St. Vincent's Hospital, 93-6 Ji-dong, Paldal-gu, Suwon, Gyeonggi-do 442-723, Republic of Korea; ^3^Samkwang Medical Laboratories, 9-60 Yangjae-dong, Seocho-gu, Seoul 137-887, Republic of Korea; ^4^Department of Laboratory Medicine, College of Medicine, The Catholic University of Korea, 62 Yeouido-dong, Yeongdeungpo-gu, Seoul 150-713, Republic of Korea; ^5^Department of Laboratory Medicine, Yonsei University Wonju College of Medicine, 20 Ilsan-ro, Ilsandong, Wonju, Gangwon-do 220-701, Republic of Korea; ^6^Department of Laboratory Medicine, Gangnam Severance Hospital, Yonsei University College of Medicine, 712 Eonjuro, Gangnam-gu, Seoul 135-720, Republic of Korea; ^7^Department of Rehabilitation, Institute of Neuromuscular Disease, Gangnam Severance Hospital, Yonsei University College of Medicine, 712 Eonjuro, Gangnam-gu, Seoul 135-720, Republic of Korea

In the paper titled “Partial Gene Deletions of* PMP22* Causing Hereditary Neuropathy with Liability to Pressure Palsies,” the affiliations of the authors should be as shown above.

